# The Weight of the First Year: The Impact of Negative Experiences for Early-Career Nurses and Midwives—A Long-Term Risk for Trauma, Burnout and Professional Attrition

**DOI:** 10.3390/nursrep16040131

**Published:** 2026-04-13

**Authors:** Helen Donovan

**Affiliations:** School of Nursing, Faculty of Health, Kelvin Grove Campus, Queensland University of Technology, Brisbane, QLD 4059, Australia; h.donovan@qut.edu.au

**Keywords:** graduate nurse, graduate midwife, long term outcomes, trauma, nursing

## Abstract

**Background**: Early-career registered nurses and midwives often encounter intense stressors that affect their physical, mental, emotional, and social well-being. While some challenges serve as valuable learning opportunities, others are traumatic and burdensome. These negative experiences can profoundly influence ongoing professional development and, for some, act as a catalyst for burnout and premature departure from the profession. **Methods**: This qualitative phenomenological study involved 51 registered nurses and midwives within their first three years of practice at an Australian hospital. The research aimed to identify the challenges faced by participants during their initial years and to explore how these experiences shaped their perceptions of ongoing practice. **Results**: Many participants in their second and third years of practice, who had difficult first-year experiences, described in detail the impact of their initial encounters on their formative nursing practice during the interviews. The challenges faced in the second and third years were often considered insignificant in comparison to the traumatic events of the first year. Some participants reported requiring long-term psychological support as a result of first year experiences and expressed uncertainty about continuing in the nursing profession. **Conclusions**: Ensuring that first-year experiences are positive, supportive, and rewarding is crucial for new nurses and midwives. When this period is marked by trauma, emotional distress can escalate, leading to burnout and an increased likelihood of professional attrition. Supporting graduates’ well-being and addressing their individual needs during these formative years is essential for workforce sustainability.

## 1. Introduction

The challenges facing graduate nurses and midwives are well documented, including a lack of professional support, feelings of unpreparedness for the clinical environment, and work overload encroaching on personal time and space [1]. These issues consistently create a diverse set of obstacles impacting the graduate’s transition, such as physical demands resulting in pain, sleep deprivation, and fatigue; mental stress from constant learning leading to cognitive saturation; and emotional demands stemming from the fear of clinical errors and the difficulties of caring for individuals in challenging circumstances [2]. Graduate nurses and midwives have reported that the intensity of work–life interference and job dissatisfaction is overwhelming, often resulting in negative affect, emotional exhaustion, and burnout [3].

Trauma is defined as “any disturbing experience that results in significant fear, helplessness, dissociation, confusion, or other disruptive feelings, intense enough to have a long-lasting negative effect on a person’s attitudes, behavior, and other aspects of functioning” [4]. Traumatic experiences in nursing and midwifery are often described from a vicarious perspective, where traumatic events are witnessed during the care of patients. Literature has recognised prolonged exposure to traumatic experiences as a catalyst for emotional stress and psychological trauma, leading to compassion fatigue and burnout [5,6]. Nurses and midwives also report direct trauma resulting from organisational and systemic incongruities, such as insufficient resources to meet patient care needs [1]. Additionally, workplace violence and subsequent sleep disturbances due to time-constrained clinical errors, along with the fear of job loss, have been reported [7].

For graduate nurses and midwives, traumatic experiences—both vicarious and direct—can overwhelm already stretched healthcare professionals who are striving to learn processes, routines, policies, and personnel in order to establish themselves as registered healthcare professionals. These stressors are well recognised and so commonplace that transition programs have been developed to actively teach strategies to assist graduate nurses and midwives to respond effectively to adverse events, by improving resilience [8] and coping abilities [9].

While the graduate year for beginning nurses and midwives can be challenging, overwhelming, and confusing, the ongoing implications for early career nurses and midwives in their second and third years have rarely been addressed. Increasing responsibilities, career progression demands and leadership role responsibilities place higher levels of expectations on the registered nurse and midwife in their second and third years of practice [10]. Unresolved personal and professional issues stemming from traumatic experiences in a nurse’s or midwife’s first year of practice could have long-term impacts, constraining confidence, performance, and career progression for these healthcare professionals.

This research explores the experiences of second- and third-year early-career nurses and midwives to identify the adversities they face and how their needs change during their transition to practice. The findings indicate that some first-year experiences in both nursing and midwifery are so traumatic and pervasive that they persist into the second and third years, presenting obstacles to confidence and professional identity. This paper reports on the perspectives of second- and third-year nurses and midwives who participated in the study, elucidating their perceived influence of first-year experiences on their subsequent professional practice.

## 2. Materials and Methods

### 2.1. Design

A qualitative phenomenological study was chosen for this research as it enabled the experiences of graduate nurses and midwives in their first three years of practice to be described, the common themes realised and the meaning behind the descriptions explored.

### 2.2. Participants

Participants in this research were early-career registered nurses and registered midwives, all employed at the same large regional hospital in Australia. Eligibility for inclusion required participants to be within their first three years of clinical practice as a registered nurse or registered midwife and they were recruited via email notification to each staff member and local signage in the clinical area. Staff were excused from the clinical area to attend an interview for data collection at their time and day of choosing. This was usually added to a meal break to reduce disruption to the clinical care provision.

### 2.3. Data Collection

Once each participant signed a consent form, data was collected through focus group or individual face-to-face semi-structured interviews, each of 15–20 min in duration. This was dependent upon the participants’ preference, ensuring participants felt comfortable and supported throughout the process. Participants were informed that their involvement was entirely voluntary and that they could withdraw from the interview or the research project at any time without consequence. In total there were forty-nine interviews with only two focus group interviews. The focus groups were undertaken with 2 members in each group at the participants’ request. The interview format was structured from a general experiential perspective [11], where the life-world experience of the participant is central to guiding and focusing on the phenomenon so that meaning can be drawn from the experience. A convergent interviewing approach was used [12], where open questioning and convergence on common points through refined questioning enabled core themes to emerge and be explored. This approach enabled the analysis to occur alongside data collection. Key concepts were drawn from verbatim transcripts and considered from both a time-working and an experiential perspective. Two researchers conducted the interviews, and validity and reliability were maintained using a systematic and documented approach to ensure consistency and to manage any issues that emerged during the data collection phase.

### 2.4. Ethics

This study was approved as low-risk research by the relevant Human Research Ethics Committee. Participant confidentiality and anonymity were maintained through the use of pseudonyms and the removal of identifying information from transcripts and reports. Data was stored securely in accordance with institutional policy, within a password-protected computer.

### 2.5. Data Analysis

To ensure the analysis was robust, transparent, and free from bias, all recorded data was transcribed verbatim using a software transcription service. Transcriptions were carefully deidentified, with only participant year level and discipline recorded in a Master Log, safeguarding confidentiality and reducing the potential for researcher bias. The data analysis followed the rigorous process outlined by Braun and Clarke [13], involving multiple readings and a close review of the data to identify meaningful descriptions. These descriptions were systematically organised into a series of generic codes. Coding was conducted with attention to consistency and transparency, allowing intentions and meanings to be catalogued into themes. These themes were then critically refined for significance, relevance, and consistency, with ongoing comparison to ensure alignment across participant groups and disciplines. For this paper, all themes related to negative experiences by graduates in their first year that carried over into their subsequent years of practice were highlighted and examined in relation to current literature, further enhancing the trustworthiness and validity of the analysis.

## 3. Results

Fifty-one participants were recruited to the research project, with all but one participant currently employed by the regional hospital at the time of the interview. There was a greater representation of first-year graduates than of those in their second year and third year (Table 1). Many areas of clinical practice were represented, including birth suite, special care nursery, emergency department, intensive care unit, rehabilitation, day surgery and general medical and surgical wards.

While the participants in the research were invited to describe their current year of nursing and midwifery experiences, many automatically reverted to describing their initial experiences in their first year of practice. For some, the need to share their experiences appeared to be cathartic and essential that the researcher understood their experiences. Those who had very negative experiences ruminated over the experience and consistently questioned the process and the outcome.

### 3.1. Thematic Summary

The experiences of the early-career nurses and midwives in this research was multifactored and multidimensional with issues resulting from a complex accumulation of psychological, emotional, interpersonal, and organisational stressors. The following themes (Table 2) represent the dominant patterns reported by graduates in their second or third year of practice as traumatic stressors experienced during their graduate year of practice.

### 3.2. Clinical Events as Traumatic Stressors

Participants in their second and third years of employment continued to experience anxiety and described in detail first-year experiences involving acute clinical incidents such as deaths, emergencies and deteriorating patient responses. Many described how they did not have the knowledge, clinical skills or support to manage these experiences. A consistent theme of being professionally alone and feeling professionally lonely emerged, impacting their ability to develop their professional identity and confidence in practice.

*I had a deteriorating patient… I was on my own banging on the window asking for help… it really floored me that no one came. After that, I was shaking on the way to work… anxious in case something else happened.*

RN3/1

A midwife interviewed at the end of their first year, who experienced an intrauterine death on their first day of practice, was still receiving psychological support to manage the experience. The midwife’s intentions to remain in the profession at the time were not clear.

*I didn’t really process the death for the first month… I had to dissociate… it made it take longer for me to actually understand what had happened.*

RM1/2

Other early-career nurses in their second and third years were still thinking about clinical experiences that negatively impacted their levels of confidence and influenced the decisions they made at the time and continue to make as more experienced registered nurses.

*I had a drug error in my first three months, so that really brought me down to ground zero. I still think about it.*

RN2/7

*I had a problem with an experienced RN demanding that I do things her way without offering evidence for it. I felt speaking up was going to rock the boat.*

RN3/6

*Delegating as a new grad RN to an EN that’s had 30 years on the same ward or even just 30 years as an EN. They don’t take it very well. So, delegation is something I really think about before doing after that experience.*

RN3/4

### 3.3. Lack of Support and Stressful Responses

The perceived and actual lack of support was significant in the participant responses.

*I was supposed to have a preceptor, but mine got sick and I was on my own for the first three months. It was really stressful.*

RN2/1

*Skill mix was never adequate and staffing ratios not equitable to provide you with the support you needed.*

RN3/8

*So, I was pretty much thrown in the deep end in my grad year, I had one supernumerary shift. And that was it on the floor. Scary, really scary. Somebody saying, here’s a key to the DD med’s [dangerous drug medication] and I’m realizing I’m not a student anymore. I’m full on responsible and have the responsibility that everybody else holds.*

RN2/1

The lack of positive support and constructive feedback was often described as undermining the beginning graduate’s ability to deliver quality care, and they stated that they had to work it out for themselves to fulfil patient care requirements.

*I YouTube everything now. I learn more this way and I don’t have to ask anyone.*

RN2/11

*I ended up having to have my own back and just fit in. I learnt the hard way I guess.*

RN2/5

*A comment from my NUM at the end of the graduate programme was, “Oh, you didn’t do anything spectacular or do anything great”. I said, can you tell me something that another Grad has done that is great for example. I haven’t done anything wrong. I haven’t killed anyone. I haven’t made any errors. At six months that feedback may have been helpful, but I was a bit jaded at the end of 12 months. I thought why are you just telling me this now?*

RN3/4

### 3.4. Discrimination and Exclusion

Both covert and overt bullying behaviours were noted, such as withholding information, ignoring the graduate’s unique needs and excluding them from team meal breaks or ward based social events. For some, the harassment was identified as intentional, aimed at undermining the graduate so that they felt invisible and powerless.

*She just says nasty stuff… I would go home upset… it was very unpleasant to watch when it happened to the next round of grads.*

RN2/10

*There weren’t any social events arranged to welcome the grads or anything. So, it’s just a matter of finding your own social life?*

RN2/3

*‘I was the only grad for three months. No one spoke to me… that was lonely’.*

RN3/4

Behaviours from healthcare professionals that intentionally undermined and discredited graduates as effective clinicians reduced the graduates’ ability to gain confidence in their practice and slowed their transition into practice.

*But yeah, they doubt you a lot. They don’t trust your clinical plan. Sometimes they’ll just tell you straight to your face. They can make you doubt your practice.*

RN2/3

*We had an educator standing in front of us, and she was mocking everyone. They’d send back the modules [educational packages for graduates] two or three times, because she wasn’t happy with things or how they were written. There was no clear feedback.*

RN2/3

### 3.5. Chronic Overload of Work

Many participants described sustained physical, cognitive and emotional overload due to high patient ratios; consistent understaffing and skill-mix issues; lack of protected learning time and no opportunity to rest, recover, or decompress. Issues such as sleep deprivation, dreading returning to work and feeling constantly unable to fulfil responsibilities were identified as key factors.

*We were allocated eleven patients… that’s a lot of patients… especially as a graduate.*

RN2/4

*I felt lost. I didn’t know where to start. There were a lot of things to do that I couldn’t do—drains etc. I was always afraid I would do something wrong.*

RN2/10

The workload was constantly underpinned by the beginning status of the graduate nurse and midwife, and with graduate midwives rotating through the specialty areas every 3 months, they were always starting over when they had only just found their feet in the previous area.

*It was stressful to rotate through the specialties… you have to start from scratch each new rotation.*

RM2/13

Educational packages referred to as ‘modules’ by the participants were designed to provide information and to test the graduate’s knowledge and understanding. For some, it was perceived as an affront to their achievements and to having met the registration requirements. This perception continued to resonate negatively in almost all of the participants’ responses.

*… seven modules to complete over twelve months. I didn’t get feedback on any of mine, so it was a waste of time. It was stuff I learned at Uni, so I learnt nothing new. I also had to do them outside work hours.*

RN2/8

*The modules were really stressful. We had seven modules with deadlines and had to finish them in our own time. They don’t count for anything, and I think that’s a bit harsh. Each was minimum twenty hours of unpaid work.*

RN2/7

Completion of the modules was often held as a prerequisite to gaining permanency. As all of the graduates were on six- or twelve-month contracts when data was gathered for this research, this was an important provision. Although for some, completion was based on workforce demands.

*They said I would not get permanency unless I did the seven modules.*

RN2/6

*They told me that I would not get a permanent position. The NUM then offered me a permanent role anyway. So, it was kinda like blackmail.*

RN2/8

### 3.6. Personal Life Disruption as Secondary Trauma

The completion of the modules continued to spill over into the graduates’ personal lives, with early-career nurses in their second and third years of practice still describing the impact that these demands had on their personal lives.

*Modules were the most stressful thing in my grad year. Having to do them in my own time made me very tired and resentful. They don’t count for anything and are compulsory. I didn’t learn anything new. I just studied that stuff in my degree.*

RN2/7

*The modules were too time-consuming. They don’t count for postgrad studies. ‘You are on the Ward, you’re trying to find your feet then you’ve got modules to do as well. I’ve got a family. There was no worklife balance during that year. Just get the modules done because they might offer a permanent position compared to someone who didn’t.*

RN3/8

Other factors that impacted their personal life as graduates, which remained challenging, were the adjustment to shift work and the impact that it had on personal time. One graduate was rostered onto night shifts in their first two weeks of their graduate year, which they recalled in detail as a challenging experience.

*Adjusting to shift work is really difficult. Everyone says that it’s going to be difficult, but you don’t understand until you’re in it.*

RN3/7

*I had nights in my first two weeks. My NUM said, I am sorry, but I have had to put you on nights. I was on my own. I haven’t done a night shift before, and I couldn’t sleep on the first day. It was sink or swim.*

RN2/7

## 4. Discussion

This study demonstrates that traumatic experiences encountered during the graduate year can exert a sustained influence on nurses’ and midwives’ perceptions of practice into their second and third years. Participants repeatedly returned to vivid recollections of early exposure to death, patient deterioration, emergency responses, interpersonal conflict, bullying, and perceived failure, despite being invited to describe their current practice. These findings suggest that the graduate year represents a period of critical vulnerability, during which adverse experiences may become psychologically encoded and shape subsequent professional identity, confidence, and engagement with practice.

Psychological literature defines trauma not solely by the objective severity of an event, but by the individual’s subjective experience of threat, helplessness, and loss of control [4]. Many participants described being left alone with critically unwell patients, feeling unheard when escalating concerns, or managing high-stakes clinical situations without adequate supervision. Such conditions reflect core trauma mechanisms, including perceived abandonment, loss of safety, dissociation, and hypervigilance, which are associated with persistent anxiety, intrusive recollections, and avoidance behaviours [14,15]. These issues are often reported by graduates as arising from feeling unsafe in their practice and unable to gain a positive sense of achievement. This not only increases the graduate’s levels of mental, emotional and physical exhaustion and sense of incompetence [2], but also leads to a sense of imposter syndrome where trust, belonging and self-doubt [10] impact the graduate and early-career nurse and midwife’s development of their professional identity and career progression.

The interaction between high clinical responsibility and professional isolation emerged as a central mechanism underpinning ongoing distress. Duchscher’s transition theory highlights that early-career clinicians experience phases of shock and disorientation and require consistent functional and emotional support to progress toward professional integration [16]. In this study, when such support was absent or unreliable, early transition experiences were perceived as overwhelming and, at times, traumatic. Rather than consolidating competence and confidence, these experiences appeared to disrupt professional development, with hesitation around delegation, decision-making, and speaking up persisting into later years of practice. These enduring negative effects have been shown to have a significant impact on nurse attrition [1], whereas a supportive work environment empowers early-career nurses to remain in their chosen profession and build professional capability and confidence [17].

Midwifery-specific findings reinforce that trauma is embedded within maternity care environments, where exposure to foetal loss, traumatic births, and high accountability is common. Participants described enduring emotional responses including fear, guilt, self-blame, and vicarious traumatisation following traumatic clinical events, consistent with contemporary midwifery literature [5,18]. Repeated exposure to trauma without adequate debriefing or organisational support increases the risk of cumulative trauma and compassion fatigue [6,19], suggesting that these responses reflect unresolved trauma rather than transient stress.

Medication errors and perceived clinical failures were also experienced as psychologically enduring events. Consistent with “second victim” literature, participants described intrusive rumination, shame, and diminished confidence, particularly within blame-oriented organisational cultures [7,20]. Hierarchical constraints further compounded distress, with participants describing an inability to speak up due to junior status or fear of reprisal. Such experiences align with moral distress theory and appeared to become embedded, influencing later confidence and leadership behaviours [21,22].

Taken together, these findings suggest that trauma in early-career nurses and midwives arises not only from discrete clinical events, but from a layered accumulation of stressors during the graduate year. When these converge, trauma may become a dominant lens through which subsequent practice is interpreted. Addressing early-career trauma therefore requires systemic, trauma-informed organisational responses that recognise the graduate year as a high-risk period for psychological harm.

### Implications for Nursing and Midwifery Practice

Nursing and midwifery services should treat the graduate year as a high-vulnerability transition period and embed trauma-informed supports that extend beyond initial orientation. Reliable preceptorship, protected supernumerary time, and clear senior escalation pathways are essential, as inadequate functional and emotional support intensifies transition distress [7,16]. Routine, structured debriefing and peer support following deaths, critical incidents, traumatic births, and errors should be standardised, reflecting evidence that second-victim effects include enduring psychological distress and that organisational support mitigates work-related impacts [3]. Leaders must actively prevent bullying and exclusion and foster psychological safety, given strong links between new-graduate mistreatment, emotional exhaustion, and poor health outcomes [1]. Workload and administrative requirements should be reviewed, reducing overload and work–life interference while strengthening supervisor support, autonomy, and fair management to promote well-being and retention [2].

## Figures and Tables

**Table 1:** Participants.

Participant	1st Year	2nd Year	3rd Year
Registered Nurse (RN)	20	16	8
Registered Midwife (RM)	4	2	1

**Table 2:** Themes.

Traumatic Stressors	Descriptors
Clinical events	Feeling undervalued and unheard Professionally alone and professionally lonely
Lack of support	Being left alone with critically unwell patients No scheduled debriefs after traumatic incidents Feeling unsafe to ask questions or seek help
Discrimination and exclusion	Snide comments, personal attacks, humiliation Age-based or gender-based bullying Being ostracised or targeted as a new graduate Constant scrutiny of decisions
Chronic overload of work	Consistent understaffing and skill-mix issues Lack of protected learning time No opportunity to rest, recover, or decompress
Personal life disruption	Insomnia, rumination, nightmares Guilt when taking sick days to recover Emotional withdrawal from relationships Inability to “turn off” after traumatic shifts

## Data Availability

Data is available from the author upon request. The data are not publicly available due to privacy and ethical reasons.

## Public Involvement Statement

The public was not involved in this research.

## Guidelines and Standards Statement

This manuscript was drafted against the SRQR for qualitative research.
